# Effects of antioxidants and MAPK inhibitors on cell death and reactive oxygen species levels in H_2_O_2_-treated human pulmonary fibroblasts

**DOI:** 10.3892/ol.2013.1216

**Published:** 2013-02-28

**Authors:** WOO HYUN PARK

**Affiliations:** Department of Physiology, Medical School, Research Institute for Endocrine Sciences, Chonbuk National University, JeonJu, Jeollabuk-do 561-180, Republic of Korea

**Keywords:** human pulmonary fibroblasts, H_2_O_2_, cell death, reactive oxygen species, mitogen-activated protein kinase

## Abstract

H_2_O_2_-induced cytotoxicity in normal human pulmonary fibroblasts (HPFs) is of interest in toxicological research since HPFs are involved in lung inflammation, fibrosis and cancer. The present study investigated the cytotoxic effects of H_2_O_2_ on normal HPFs in relation to reactive oxygen species (ROS) and mitogen-activated protein kinases (MAPKs) using the well-known antioxidants N-acetyl cysteine (NAC) and propyl gallate (PG), as well as MAPK inhibitors. Treatment with 50 *μ*M H_2_O_2_ inhibited the growth of the HPFs by ∼45% in 24 h. H_2_O_2_ induced cell death via apoptosis and triggered the loss of mitochondrial membrane potential (MMP; Δψ_m_) in the HPFs. H_2_O_2_ also increased the ROS levels, including O_2_^•−^, in the HPFs and induced glutathione (GSH) depletion. NAC and PG attenuated the death of the HPFs and the loss of MMP (Δψ_m_) through the use of H_2_O_2_. NAC decreased the ROS levels in the H_2_O_2_-treated HPFs and PG markedly prevented an increase in O_2_^•−^ levels in these cells. However, PG alone induced cell death in the HPF control cells and increased the ROS levels in these cells. None of the MAPK (MEK, JNK and p38) inhibitors affected cell growth inhibition or cell death by H_2_O_2_. In addition, these inhibitors did not significantly affect the ROS levels and GSH depletion in the H_2_O_2_-treated HPFs. In conclusion, H_2_O_2_ induced growth inhibition and cell death in the HPFs via GSH depletion. NAC and PG attenuated H_2_O_2_-induced HPF cell death but each regulated the ROS levels in a different manner. Treatment with MAPK inhibitors did not affect cell death or the ROS levels in the H_2_O_2_-treated HPFs.

## Introduction

Reactive oxygen species (ROS) are mainly comprised of hydrogen peroxide (H_2_O_2_), superoxide anions (O_2_^•−^) and hydroxyl radicals (^•^OH), which affect numerous cellular processes, including metabolism, differentiation and cell proliferation and death, by regulating critical signaling pathways (1,2). Unlike other ROS, H_2_O_2_ is capable of freely diffusing through biological membranes the width of several cells prior to reacting with specific molecular targets. ROS are mostly generated during the process of mitochondrial respiration and by specific oxidases, including nicotine adenine diphosphate (NADPH) oxidase and xanthine oxidase (3). The main metabolic pathways use superoxide dismutases, which metabolize O_2_^•−^ to H_2_O_2_(4). Further metabolism by catalase or glutathione (GSH) peroxidase yields O_2_ and H_2_O (5). Oxidative stress occurs through an increase in ROS levels and/or a decrease in cellular antioxidants, and leads to cell death (6–8). Exogenous H_2_O_2_ is frequently used as a representative ROS in modeling and inducing oxidative stress.

Three major groups of mitogen-activated protein kinases (MAPKs) exist: extracellular signal-regulated kinase (ERK1/2), c-Jun N-terminal kinase/stress-activated protein kinase (JNK/SAPK) and p38 (9). MAPKs are involved in crucial signaling pathways in cell proliferation, differentiation and cell death in response to various signals produced by growth factors, hormones and cytokines, as well as genotoxic and oxidative stressors (9,10). Each MAPK pathway has comparatively varied upstream activators and unambiguous substrates (11). Abundant evidence has demonstrated that JNK and p38 are activated by ROS or mild oxidative shifts in the intracellular thiol/disulfide redox state, initiating processes associated with apoptosis (12,13). ROS provoke ERK phosphorylation and also stimulate the ERK pathway (14). In the majority of instances, ERK activation has a pro-survival effect rather than a pro-apoptotic effect (15). In addition, MAPK pathways are also activated by the direct inhibition of MAPK phosphatases by the ROS. Since the differing and opposing effects on MAPKs are caused by various ROS in the cells, the correlation between ROS and MAPKs requires further clarification, particularly with regard to the signaling associated with cell survival and death.

Cultured normal human cells are invaluable biological models for mechanistic studies of oxidative stress. H_2_O_2_-induced cytotoxicity in normal fibroblast cells *in vitro* may be of interest in toxicological research with regard to the toxic potential of exogenous H_2_O_2_ in human pulmonary fibroblasts (HPFs) since HPFs are closely involved in lung inflammation, fibrosis and cancer. However, the toxicological mechanism of the effects of exogenous H_2_O_2_ on normal HPFs remains unknown with regard to MAPKs. The present study investigated the effects of the well-known antioxidants N-acetyl cysteine (NAC) and propyl gallate (PG), as well as the MAPK inhibitors, on H_2_O_2_-treated HPFs in relation to cell growth and death and the ROS and GSH levels.

## Materials and methods

### Cell culture

HPFs purchased from PromoCell GmbH (Heidelberg, Germany) were maintained in a humidified incubator at 37°C with 5% CO_2_. The HPFs were cultured in RPMI-1640 supplemented with 10% (v/v) fetal bovine serum (FBS) and 1% (v/v) penicillin-streptomycin (GIBCO BRL, Grand Island, NY, USA). The HPFs were grown in 100-mm plastic tissue culture dishes (Nunc, Roskilde, Denmark) and harvested with trypsin-EDTA solution while in the logarithmic growth phase. The HPFs between passages four and eight were used. The study was approved by the Ethics Committee of Chonbuk National University, Jeonju, Republic of Korea.

### Reagents

H_2_O_2_, NAC and PG were purchased from Sigma-Aldrich Chemical Company (St. Louis, MO, USA). The NAC was dissolved in buffer [20 mM HEPES (pH 7.0)], while the PG was dissolved in ethanol at 200 mM as a stock solution. JNK inhibitors (SP600125), MEK inhibitors (PD98059) and p38 inhibitors (SB203580) were purchased from Calbiochem (San Diego, CA, USA). All the inhibitors were dissolved in DMSO at 10 mM as stock solutions. The HPFs were pretreated with 2 mM NAC, 400 *μ*M PG or 10 *μ*M MAPK inhibitors for 1 h prior to treatment with H_2_O_2_. Ethanol (0.2%) and DMSO (0.2%) were used as control vehicles and did not affect cell growth or death.

### Cell growth and cell number assays

The changes in cell growth in the HPFs were indirectly determined by measuring the 3-(4,5-dimethylthiazol-2-yl)-2,5-diphenyltetrazolium bromide (MTT; Sigma-Aldrich Chemical Company) dye absorbance. In brief, 4×10^4^ cells per well were seeded in 96-well microtiter plates (Nunc). Following exposure to 50 *μ*M H_2_O_2_, with or without 2 mM NAC, 400 *μ*M PG or 10 *μ*M MAPK inhibitors for 24 h, 20 *μ*l MTT solution (2 mg/ml in PBS) was added to each well of the 96-well plates. The plates were incubated for an additional 4 h at 37°C. The media in the plates were withdrawn by pipetting and 200 *μ*l DMSO was added to each well to solubilize the formazan crystals. The optical density was measured at 570 nm using a microplate reader (Synergy™ 2; BioTek Instruments Inc., Winooski, VT, USA).

### Annexin V-fluorescein isothiocyanate (FITC) staining for cell death detection

Apoptosis was determined by staining cells with annexin (Invitrogen Corporation, Camarillo, CA, USA; Ex/Em = 488 nm/519 nm). In brief, 1×10^6^ cells were incubated in a 60-mm culture dish (Nunc) with 50 *μ*M H_2_O_2_, with or without 2 mM NAC, 400 *μ*M PG or 10 *μ*M MAPK inhibitors for 24 h. The cells were washed twice with cold PBS, then resuspended in 500 *μ*l binding buffer (10 mM HEPES/NaOH, pH 7.4; 140 mM NaCl; 2.5 mM CaCl_2_) at a concentration of 1×10^6^ cells/ml. Annexin V-FITC (5 *μ*l) was then added to the cells, which were analyzed with a FACStar flow cytometer (Becton Dickinson, Franklin Lakes, NJ, USA).

### Measurement of mitochondrial membrane potential (MMP; Δψ_m_)

MMP (Δψ_m_) levels were measured with a rhodamine 123 fluorescent dye (Sigma-Aldrich Chemical Company; Ex/Em = 485 nm/ 535 nm). In brief, 1×10^6^ cells were incubated in a 60-mm culture dish (Nunc) with 50 *μ*M H_2_O_2_, with or without 2 mM NAC, 400 *μ*M PG or 10 *μ*M MAPK inhibitors for 24 h. The cells were washed twice with PBS and incubated with the rhodamine 123 (0.1 *μ*g/ml) at 37°C for 30 min. Rhodamine 123 staining intensity was determined with a FACStar flow cytometer (Becton Dickinson). Rhodamine 123-negative cells indicated the loss of MMP (Δψ_m_) in cells.

### Detection of intracellular ROS levels

Intracellular ROS such as H_2_O_2_, ^•^OH and ONOO^•^ were detected using an oxidation-sensitive fluorescent probe dye, 2′,7′-dichlorodihydrofluorescein diacetate (H_2_DCFDA; Ex/Em = 495 nm/529 nm; Invitrogen Molecular Probes, Eugene, OR, USA). H_2_DCFDA is poorly selective for O_2_^•−^. By contrast, dihydroethidium (DHE; Ex/Em = 518 nm/605 nm; Invitrogen Molecular Probes) is highly selective for O_2_^•−^ among all the ROS. In brief, 1×10^6^ cells were incubated in a 60-mm culture dish (Nunc) with 50 *μ*M H_2_O_2_, with or without 2 mM NAC, 400 *μ*M PG or 10 *μ*M MAPK inhibitors for 24 h. The cells were then incubated with 20 *μ*M H_2_DCFDA or dihydroethidium (DHE) at 37°C for 30 min. The fluorescence of DCF and DHE was detected using a FACStar flow cytometer (Becton Dickinson). The ROS and O_2_^•−^ levels were expressed as the mean fluorescence intensity (MFI), which was calculated by CellQuest software (Becton Dickinson).

### Detection of the intracellular GSH

The cellular GSH levels were analyzed using a 5-chloromethylfluorescein diacetate dye (CMFDA, Ex/Em = 522 nm/595 nm; Invitrogen Molecular Probes). In brief, 1×10^6^ cells were incubated in a 60-mm culture dish (Nunc) with 50 *μ*M H_2_O_2_, with or without 2 mM NAC, 400 *μ*M PG or 10 *μ*M MAPK inhibitors for 24 h. The cells were then incubated with 5 *μ*M CMFDA at 37°C for 30 min. The CMF fluorescence intensity was determined using a FACStar flow cytometer (Becton Dickinson). CMF-negative (GSH-depleted) cells were expressed as the percent of CMF^−^ cells.

### Statistical analysis

The results represent the mean of at least two independent experiments (mean ± SD). The data were analyzed using Instat software (GraphPad Prism4; GraphPad Software, San Diego, CA, USA). The Student’s t-test and a one-way analysis of variance (ANOVA) with post hoc analysis, using Tukey’s multiple comparison, were applied to the parametric data. P<0.05 was considered to indicate a statistically significant difference.

## Results

### Effects of NAC and PG on cell growth and death and MMP (Δψ_m_) levels in H_2_O_2_-treated HPFs

**Figure 1:**
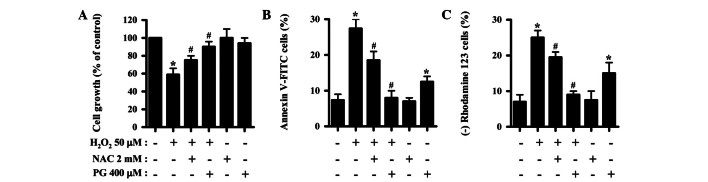
Effects of NAC and PG on cell growth and death and MMP (Δψ_m_) levels in the H_2_O_2_-treated HPFs. Exponentially-growing HPFs were treated with 50 *μ*M H_2_O_2_ for 24 h following a 1 h pre-incubation with 2 mM NAC or 400 *μ*M PG. (A) Cellular growth changes in HPFs, as assessed by MTT assays. (B) Percentages of annexin V-FITC-positive cells, as measured by flow cytometry. (C) Percentages of rhodamine 123-negative [MMP (Δψ_m_) loss] cells. ^*^P<0.05 compared with the control group. ^#^P<0.05 compared with cells treated with H_2_O_2_ only. NAC, N-acetyl cysteine; PG, propyl gallate; MMP, mitochondrial membrane potential; HPF, human pulmonary fibroblast; MTT, 3-(4,5-dimethylthiazol-2-yl)-2,5-diphenyltetrazolium bromide; V-FITC, Annexin V-fluorescein isothiocyanate.

The effects of NAC and PG on cell growth and death and MMP (Δψ_m_) levels were investigated in H_2_O_2_-treated HPFs at 24 h using MTT assays. A concentration of 50 *μ*M H_2_O_2_ was used as an optimal dose in this experiment; this inhibited the growth of the HPFs by ∼45% in 24 h (Fig. 1A). NAC and PG significantly reduced the growth inhibition caused by H_2_O_2_, with PG showing a more marked effect (Fig. 1A). H_2_O_2_ increased the percentage of annexin V-FITC stained cells among the HPFs, indirectly indicating that the HPF cell death caused by H_2_O_2_ occurred via apoptosis (Fig. 1B). NAC and PG significantly reduced the number of annexin V-FITC-positive cells in the H_2_O_2_-treated HPFs, while PG completely prevented the HPF cell death caused by H_2_O_2_ (Fig. 1B). Notably, PG alone increased the number of annexin V-FITC-positive cells among the control HPFs (Fig. 1B). Since cell death is closely associated with the collapse of MMP (Δψ_m_) (16), the effect of H_2_O_2_ on MMP (Δψ_m_) in the HPFs was assessed using a rhodamine 123 dye. Treatment with 50 *μ*M H_2_O_2_ significantly induced the loss of MMP (Δψ_m_) in the HPFs (Fig. 1C). NAC and PG attenuated the loss of MMP (Δψ_m_) caused by H_2_O_2_, while PG totally prevented this loss (Fig. 1C). Similar to the number of annexin V-FITC-positive cells, PG also increased the number of cells that lost MMP (Δψ_m_) among the control HPFs (Fig. 1C).

### Effects of NAC and PG on intracellular ROS and GSH levels in H_2_O_2_-treated HPFs

**Figure 2:**
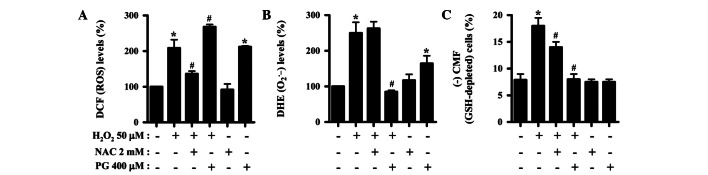
Effects of NAC and PG on intracellular ROS and GSH levels in H_2_O_2_-treated HPFs. Exponentially-growing HPFs were treated with 50 *μ*M H_2_O_2_ for 24 h following a 1 h pre-incubation with 2 mM NAC or 400 *μ*M PG. ROS levels in the HPFs were measured using a FACStar flow cytometer. (A) DCF (ROS) levels (%) in the HPFs compared with the control cell group. (B) DHE (O_2_^•^^−^) levels (%) in the HPFs compared with the control cell group. (C) CMF^−^ (GSH-depleted) cells (%) in the HPFs. ^*^P<0.05 compared with the control group. ^#^P<0.05 compared with cells treated with H_2_O_2_ only. NAC, N-acetyl cysteine; PG, propyl gallate; ROS, reactive oxygen species; HPF, human pulmonary fibroblast; GSH, glutathione; DCF, 2′,7′-dichlorodihydrofluorescein diacetate; DHE, dihydroethidium; CMF, 5-chloromethylfluorescein diacetate.

H_2_DCFDA and DHE dyes were used to assess intracellular ROS levels in the H_2_O_2_-treated HPFs. As shown in Fig. 2A, the levels of ROS (DCF), such as H_2_O_2,_ were increased in the HPFs treated with 50 *μ*M at 24 h. NAC significantly suppressed the increased ROS levels in the H_2_O_2_-treated HPFs, whereas PG enhanced the increase in ROS levels caused by H_2_O_2_ (Fig. 2A). Moreover, PG alone markedly increased the ROS (DCF) levels in the control HPFs (Fig. 2A). When the intracellular O_2_^•−^ levels were assessed in the H_2_O_2_-treated HPFs, the level of DHE fluorescence dye, which specifically indicates O_2_^•−^ accumulation in cells, was increased at 24 h (Fig. 2B). While NAC did not alter the O_2_^•−^ level in the H_2_O_2_-treated HPFs, PG entirely attenuated the increase in these cells (Fig. 2B). However, PG alone increased the O_2_^•−^ level in the control HPFs (Fig. 2B). When the intracellular GSH levels were measured in the H_2_O_2_-treated HPFs using a CMFDA dye, 50 *μ*M H_2_O_2_ was shown to increase the number of GSH-depleted cells in the HPFs at 24 h (Fig. 2C). NAC and PG significantly reduced the number of GSH-depleted cells in the H_2_O_2_-treated HPFs, while PG completely prevented the GSH depletion (Fig. 2C).

### Effects of MAPK inhibitors on cell growth and death and MMP (Δψ_m_) levels in H_2_O_2_-treated HPFs

**Figure 3:**
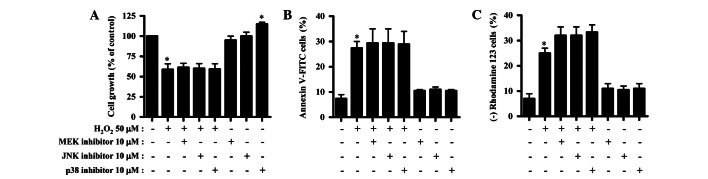
Effects of MAPK inhibitors on cell growth and death and MMP (Δψ_m_) levels in H_2_O_2_-treated HPFs. Exponentially-growing HPFs were treated with 50 *μ*M H_2_O_2_ for 24 h following a 1 h pre-incubation with each MAPK inhibitor. (A) Cellular growth changes in the HPFs, as assessed by MTT assays. (B) Percentages of annexin V-FITC-positive cells, as measured by FACStar flow cytometry. (C) Percentages of rhodamine 123-negative [MMP (Δψ_m_) loss] cells. ^*^P<0.05 compared with the control group. ^#^P<0.05 compared with cells treated with H_2_O_2_ only. MAPK, mitogen-activated protein kinase; MEK, MAP/ERK kinase; JNK, c-Jun N-terminal kinase; MMP, mitochondrial membrane potential; HPF, human pulmonary fibroblast; MTT, 3-(4,5-dimethylthiazol-2-yl)-2,5-diphenyltetrazolium bromide; V-FITC, Annexin V-fluorescein isothiocyanate.

The effect of the MAPK inhibitors on cell growth and death and MMP (Δψ_m_) levels in the H_2_O_2_-treated HPFs was evaluated. Based on previous studies (17,18), 10 *μ*M of each MAPK inhibitor was used as an optimal dose in the present study. None of the MAPK inhibitors affected the growth inhibition caused by H_2_O_2_ (Fig. 3A). p38 inhibitor alone increased the growth of control HPFs (Fig. 3A). Additionally, none of the MAPK inhibitors affected the number of annexin V-stained cells among the H_2_O_2_-treated or -untreated HPFs (Fig. 3B). All the MAPK inhibitors appeared to enhance the loss of MMP (Δψ_m_) in the H_2_O_2_-treated HPFs (Fig. 3C).

### Effects of MAPK inhibitors on intracellular ROS and GSH levels in H_2_O_2_-treated HPFs

**Figure 4:**
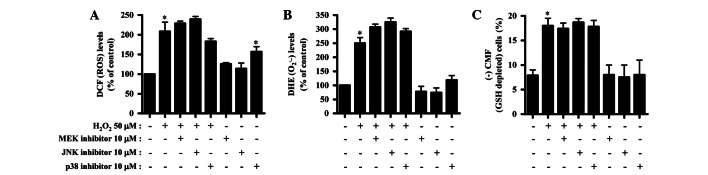
Effects of MAPK inhibitors on intracellular ROS and GSH levels in H_2_O_2_-treated HPFs. Exponentially-growing HPFs were treated with 50 *μ*M H_2_O_2_ for 24 h following a 1 h pre-incubation with each MAPK inhibitor. ROS levels in the HPFs were measured using a FACStar flow cytometer. (A) DCF (ROS) levels (%) in the HPFs compared with the control cell group. (B) DHE (O_2_^•^^−^) levels (%) in the HPFs compared with the control cell group. (C) CMF^−^ (GSH-depleted) cells (%) in the HPFs. ^*^P<0.05 compared with the control group. ^#^P<0.05 compared with cells treated with H_2_O_2_ only. MAPK, mitogen-activated protein kinase; MEK, MAP/ERK kinase; JNK, c-Jun N-terminal kinase; ROS, reactive oxygen species; HPF, human pulmonary fibroblast; GSH, glutathione; DCF, 2′,7′-dichlorodihydrofluorescein diacetate; DHE, dihydroethidium; CMF, 5-chloromethylfluorescein diacetate.

The changes in intracellular ROS levels in the H_2_O_2_ and/or each MAPK inhibitor-treated HPF were assessed. As shown in Fig. 4A, the MEK and JNK inhibitors only marginally enhanced the ROS levels in the H_2_O_2_-treated HPFs, whereas the p38 inhibitor appeared to decrease the ROS levels (Fig. 4A). In addition, all the MAPK inhibitors marginally intensified the O_2_^•−^ level increase caused by H_2_O_2_ (Fig. 4B). The p38 inhibitor alone increased the ROS levels, including the O_2_^•−^ level, in the HPF control cells (Fig. 4A and B). Moreover, none of the MAPK inhibitors affected the number of GSH-depleted cells in the H_2_O_2_-treated or untreated HPFs (Fig. 4C).

## Discussion

HPFs are pathophysiologically involved in lung inflammation, fibrosis and cancer since these cells synthesize extracellular matrix and collagen to maintain the structural and functional integrity of the lung. The present study focused on elucidating the cytotoxic effect of exogenous H_2_O_2_ on cell growth and death in normal HPFs, in relation to ROS and MAPKs. Treatment with 50 *μ*M H_2_O_2_ inhibited the growth of HPFs by ∼45% in 24 h. H_2_O_2_ increased the number of annexin V-FITC-positive cells among the HPFs, indicating that H_2_O_2_-induced HPF cell death occurred via apoptosis. An increase in caspase-3 activity was also observed in the H_2_O_2_-treated HPFs (data not shown). In addition, H_2_O_2_ triggered the loss of MMP (Δψ_m_) in the HPFs. The level cells with MMP (Δψ_m_) loss appeared to be similar to that of the annexin V stained cells, suggesting that cell death caused by H_2_O_2_ was markedly correlated with the collapse of MMP (Δψ_m_).

ROS toxicity in cells is generally mediated by ^•^OH (19). H_2_O_2_ and O_2_^•−^ are the main ROS involved in the cell signaling pathways. According to the present results, the ROS levels, including those of O_2_^•−^, were significantly increased in the HPFs treated with H_2_O_2_. Since 50 *μ*M H_2_O_2_ induced cell death and MMP (Δψ_m_) loss in the HPFs, it is possible that exogenous H_2_O_2_ generates O_2_^•−^ by damaging the mitochondria and that H_2_O_2_ and O_2_^•−^ may be efficiently converted into toxic ^•^OH via the Fenton reaction, resulting in the death of the HPFs. It is also possible that H_2_O_2_ activates oxidases, such as NADPH oxidase and xanthine oxidase, in HPFs to generate O_2_^•−^. In th epresent study, NAC attenuated the growth inhibition and cell death of the H_2_O_2_-treated HPFs and also significantly attenuated the MMP (Δψ_m_) loss in these cells. NAC markedly decreased the ROS (DCF) levels in the H_2_O_2_-treated HPFs. However, NAC did not reduce the increased O_2_^•−^ (DHE) level caused by H_2_O_2_, suggesting that NAC did not block the O_2_^•−^ generation pathway induced by exogenous H_2_O_2_. In addition, the O_2_^•−^ level in the HPFs co-treated with H_2_O_2_ and NAC did not appear to be correlated with HPF cell death.

PG, as a synthetic antioxidant, exerts a variety of effects on tissues and cells. For example, PG is an efficient protector of liver cells against lipid peroxidation by oxygen radicals (20). By contrast, PG has pro-oxidant properties (21,22). The anti-oxidative and cytoprotective properties of PG may change to pro-oxidative, cytotoxic and genotoxic properties in the presence of Cu(II) (23). The present results demonstrated that PG alone marginally inhibited the growth of the HPFs and induced cell death accompanied by the loss of MMP (Δψ_m_). In addition, PG increased the ROS levels, including those of O_2_^•−^, in the HPFs. Thus, it is possible that PG, as a pro-oxidant, is able to directly generate mitochondrial O_2_^•−^ in the HPFs by impairing mitochondrial function, consequently leading to HPF cell death via oxidative stress. Similarly, it has been reported that PG causes cytotoxic effects in isolated rat hepatocytes by causing mitochondrial damage (24) and that it also increases mitochondrial O_2_^•^^−^ levels in HeLa cells (25). Notably, PG markedly attenuated the growth inhibition and cell death in the H_2_O_2_-treated HPFs and also prevented MMP (Δψ_m_) loss in these cells. Moreover, PG completely abrogated the O_2_^•−^ (DHE) level increase caused by H_2_O_2_. Therefore, PG appeared to protect the HPFs against exogenous H_2_O_2_ by protecting the mitochondria. However, PG increased the ROS (DCF) levels in the H_2_O_2_-treated HPFs. Numerous studies, including the present study, support the hypothesis that PG has a role as an antioxidant (20,26,27) or as a pro-oxidant (21,22), depending on various conditions, such as the cell culture media, the co-treated drugs and the cell types. PG is likely to have differing effects on the levels of the different ROS in the cells. Further studies are required to elucidate the exact roles of the types of ROS in PG-treated HPFs.

In the present study, the MEK inhibitor, which is likely to inactivate ERK, did not affect growth inhibition or cell death in the H_2_O_2_-treated HPFs. Thus, H_2_O_2_ did not directly regulate the signaling associated with ERK in the HPFs to induce their growth inhibition and death. In addition, the JNK and p38 MAPKs, which are generally associated with cell death (12,13), were not likely to be affected by H_2_O_2_ in the HPFs since none of the inhibitors affected the growth inhibition and cell death caused by H_2_O_2_. The p38 inhibitor alone increased the growth of the control HPFs, suggesting that p38 signaling is involved in the basal level of HPF growth. With regard to MMP (Δψ_m_), all the MAPK inhibitors marginally increased the loss of MMP (Δψ_m_) in the H_2_O_2_-treated HPFs, indicating that the dysregulation of these MAPK signalings enhanced the loss in these cells. Moreover, the MAPK inhibitors marginally, but not significantly, affected the ROS levels, including that of O_2_^•−^, in the H_2_O_2_-treated HPFs. The p38 inhibitor increased the ROS levels in the HPF control cells regardless of the level of cell death, while the other inhibitors mildly affected the ROS levels, including that of O_2_^•−^. Thus, the MAPK signalling in the H_2_O_2_-treated and -untreated HPFs did not meaningfully change the redox state to affect HPF death.

GSH is a key cellular non-protein antioxidant, which reduces H_2_O_2_ to H_2_O using GSH peroxidase (28). The intra-cellular GSH content has a significant effect on anticancer drug-induced apoptosis, indicating that apoptotic effects are inversely proportional to the GSH content (29,30). Similarly, in the present study, H_2_O_2_ increased the number of GSH-depleted cells in the HPFs. NAC and PG demonstrated anti-apoptotic effects on the H_2_O_2_-treated HPFs, significantly suppressing the GSH depletion in these cells. In addition, none of the MAPK inhibitors affected the GSH depletion in the H_2_O_2_-treated HPFs. Therefore, the intracellular GSH content appears to be a decisive factor in HPF cell death. However, PG alone induced cell death in the HPF control cells but it did not significantly induce GSH depletion,, suggesting that PG-induced HPF cell death is not highly associated with changes in the GSH level.

In conclusion, H_2_O_2_ induced growth inhibition and death in the HPFs via GSH depletion. NAC and PG attenuated H_2_O_2_-induced HPF cell growth inhibition and death, but each antioxidant affected the ROS levels, including that of O_2_^•−^, differently in the H_2_O_2_-treated and -untreated HPFs. Treatment with MAPK inhibitors did not affect cell death or the ROS levels in the H_2_O_2_-treated HPFs. The present data provide useful information concerning the toxicological effect of exogenous H_2_O_2_ on normal HPFs with regard to ROS and MAPKs.

## Abbreviations:

HPF: human pulmonary fibroblast;
ROS: reactive oxygen species;
MAPK: mitogen-activated protein kinase;
MEK: MAP/ERK kinase;
ERK: extracellular signal-regulated kinase;
JNK: c-Jun N-terminal kinase;
MMP (Δψ_m_): mitochondrial membrane potential;
MTT: 3-(4,5-dimethylthiazol-2-yl)-2,5-diphenyltetrazolium bromide;
FITC: fluorescein isothiocyanate;
H_2_DCFDA: 2′,7′-dichloro dihydrofluorescein diacetate;
DHE: dihydroethidium;
GSH: glutathione;
CMFDA: 5-chloromethylfluorescein diacetate;
NAC: N-acetyl cysteine;
PG: propyl gallate
